# A risk score model based on lipid metabolism-related genes could predict response to immunotherapy and prognosis of lung adenocarcinoma: a multi-dataset study and cytological validation

**DOI:** 10.1007/s12672-023-00802-3

**Published:** 2023-10-24

**Authors:** Yangyang Lei, Boxuan Zhou, Xiangzhi Meng, Mei Liang, Weijian Song, Yicheng Liang, Yushun Gao, Minghui Wang

**Affiliations:** 1 Sun Yat-Sen Memorial Hospital, Sun Yat-Sen University, Guangzhou, China; 2 Department of Thoracic Surgery, National Cancer Center/National Clinical Research Center for Cancer/Cancer Hospital, Chinese Academy of Medical Sciences and Peking Union Medical College, Beijing, China; 3 Department of Thoracic Surgery, Sun Yat-Sen Memorial Hospital, Sun Yat-Sen University, 107 Yanjiang West Road, Guangzhou, 510120 China; 4 Guangdong Provincial Key Laboratory of Malignant Tumor Epigenetics and Gene Regulation, Sun Yat-Sen Memorial Hospital, Sun Yat-Sen University, Guangzhou, China

**Keywords:** Lipid metabolism, Lung adenocarcinoma, PTDSS1, Molecular blueprint, Immune features

## Abstract

**Background:**

Lipid metabolism is a key factor in tumorigenesis and drug resistance, and models related to lipid metabolism have shown potential to predict survival and curative effects of adjuvant therapy in various cancers. However, the relationship between lipid metabolism and prognosis and treatment response of lung adenocarcinoma (LUAD) are still unclear.

**Methods:**

We enrolled seven bulk RNA-sequence datasets (GSE37745, GSE19188, GSE30219, GSE31547, GSE41271, GSE42127, and GSE72094) from the GEO database and one single-cell RNA-sequencing dataset (GSE117570) from the TISCH2 database. Non-negative matrix factorization (NMF) was utilized to construct the risk score model based on lipid score calculated by GSVA algorithm. Phs000452.v3, PMID: 26359337, PMID: 32472114, PRJEB23709 datasets were used to test the response to immunotherapy. Drug sensitivity analysis was assessed according to the GDSC database, and immunotherapy response was evaluated using the Wilcoxon test. Cellular function assays including clone formation, EDU assays and flow cytometry were implemented to explore the phenotype alteration caused by the knockdown of PTDSS1, which is one of key gene in risk score model.

**Results:**

We analyzed both bulk and single-cell RNA sequencing data to establish and validate a risk score model based on 18 lipid metabolism-related genes with significant impact on prognosis. After divided the patients into two groups according to risk score, we identified differences in lipid-related metabolic processes and a detailed portrait of the immune landscapes of high- and low-risk groups. Moreover, we investigated the potentials of our risk score in predicting response to immunotherapy and drug sensitivity. In addition, we silenced PTDSS1 in LUAD cell lines, and found that the proliferation of the cells was weakened, and the apoptosis of the cells was increased.

**Conclusion:**

Our study highlights the crucial roles of lipid metabolism in LUAD and provides a reliable risk score model, which can aid in predicting prognosis and response to immunotherapy. Furthermore, we investigated the roles of PTDSS1 in LUAD carcinogenesis, which showed that PTDSS1 regulated proliferation and apoptosis of LUAD cells.

**Supplementary Information:**

The online version contains supplementary material available at 10.1007/s12672-023-00802-3.

## Introduction

Lung cancer emerges as the leading cause of global cancer-related incidence and mortality [1]. Among all lung cancer subtypes, lung adenocarcinoma (LUAD) proved to be the most common pathological subtype in all cases, and about 40% of lung cancers were LUAD [2, 3]. LUAD appears to be difficult to conquer due to its rapid progression and susceptibility to early relapse. It exhibits significant heterogeneity and a complex tumor microenvironment [4]. Although the application of immunotherapy and molecular targeted therapy has improved the overall survival rate (OS), there is still room for further improvement in the prognosis of LUAD. The development of novel biomarkers and reliable models for predicting treatment response can aid in improving the prognosis of LUAD and provide customized adjuvant therapy strategies.

Lipid metabolism reprogramming proves to be a hallmark of various malignant tumors [5, 6]. One striking feature of tumor cells is their aberrant uptake of lipids and cholesterol, which promotes their proliferation and division [7]. Reprogramming of lipid metabolism also constitutes a potential mechanism underlying drug resistance in anti-tumor therapy. Accordingly, many studies has already investigated the role of lipid-associated phenotypic indicators in a variety of cancers. For instance, a specific gene signature related to fatty acid metabolism was found to predict OS. Moreover, the same gene tag showed certain potency in predicting the response to chemotherapy and immunotherapy in patients suffering colorectal cancer [8]. Furthermore, Wu et al*.* recognized a lipid metabolism-related phenotype in glioma, which leaded to the development of a lipid metabolism gene signature to predict OS of patients suffering advanced glioma [9]. However, there are currently no systematic studies characterizing a superior genetic model based on lipid metabolism in LUAD to predict prognosis and antitumor response. This deserves further in-depth study.

In this study, we implemented a series of bioinformatic methods to investigate the features of lipid metabolism alterations in LUAD using bulk RNA-sequencing and single cell RNA-sequencing (scRNA-seq) profiling data from multiple databases. Subsequently, we established and validated a lipid metabolism-related gene signature for predicting LUAD prognosis. We examined the differences in lipid metabolism and immune landscapes between the low- and high-risk groups and constructed a risk score model called “Lipid-score” to predict LUAD prognosis. In addition, we investigated the potential of our Lipid-score to predict response to immunotherapy and drug sensitivity in LUAD patients. We further investigated the role of PTDSS1, namely a key gene in the model, using multiple cellular experiments. Our findings provide a promising potential biomarker for LUAD patients with high prognostic value and guidance for individualized treatment.

## Methods

### Data collection and processing

Fragments per kilobase of exon model per Million mapped fragments expression matrix of LUAD was obtained from GDC (https://portal.gdc.cancer.gov) portal. We have enrolled high-throughput sequencing transcriptome dataset GSE37745, GSE19188, GSE30219, GSE31547, GSE41271, GSE42127, GSE72094 (BULK transcriptome sequencing data for verification), phs000452.v3, PMID: 26359337, PMID: 32472114, PRJEB23709 (BULK sequencing data on the transcriptome level for verification of the response to immunotherapy) from the GEO (https://www.ncbi.nlm.nih.gov/geo/) dataset. GSE117570 (scRNA-seq data of LUAD) was obtained from TISCH2 database (http://tisch.comp-genomics.org/). All genes must be present in at least 3 cells, and at least 200 genes must be expressed in each cell. UMIs were retained at 500–6500 according to distribution, and the percentage of mitochondrial readings was less than 80%. The scRNA-seq data are transformed into UMI transcription matrix and cell information matrix, which are subsequently directed into “Seraut” package for further processing and subsequent analysis. LogNormalize is utilized for the standardization of the data.

### Cell culture and cell transfection

Two cell lines of LUAD were used in this study. Both the A549 and NCI-H1975 were brought from Cyagen Biosciences (Suzhou, China). The cells were cultured in complete medium, which consisted of 90% DMEM (Gibco, USA), 10% FBS (Hyclone, USA), and 1% penicillin–streptomycin (Gibco, USA). Optical microscopy was utilized to observe the morphology of the cells. For cell transfection, A549 and NCI-H1975 were transfected with PTDSS1 knockdown siRNA. We used the 6-well plates for cell inoculation in the condition of a confluence reaching approximately 70–80%.

### Differential expression analysis and enrichment analysis

Deseq2 was utilized to carry out the differential expression analysis in bulk transcriptome data. In the single-cell transcriptome data, FindAllMarkers was used to calculate the difference between each cell subgroup and other cells, the P-value < 0.05 and the absolute value of log_2_FC > 0.5 were selected as the threshold. GSEA pathway enrichment analysis was performed using R package “clusterProfiler”. GSVA was used to calculate the enrichment score of single tumor sample in hallmark pathway.

### Development of gene signature in lipid metabolism in LUAD

Conservative subtypes of LUAD were constructed using non-negative matrix factorization (NMF). The optimal number of the grouping status was determined to be 3 by decreasing the maximum co-occurrence correlation coefficient. The model construction was based on the prognostic differentiation of lipid molecule metabolism gene set in TCGA-LUAD, and the genes with significant prognostic efficacy were selected. Based on the rank between samples, the scores are calculated by the GSVA algorithm.

### Immune infiltration landscape

R package IOBR (0.99.9) and ssGSEA was used to evaluate the abundance levels of infiltrated immune cells. IOBR (0.99.9) is based on XCELL and EPIC immune cell infiltration algorithm [10].

### Drug sensitivity analysis

The expression levels of LUAD cell lines were procured by extracting from the GDSC database. The Lipid-score of each cell line was calculated and grouped based on the median. Combined with the AUC and IC50 data of the drugs, which were reported to possess the treating potency, Spearman correlation analysis was used to calculate their correlation with Lipid-score.

### Immunotherapy response

Phs000452.v3, PMID: 26359337, PMID: 32472114, PRJEB23709 datasets were used to test the efficacy of immunotherapy. We grouped the patients according to the score calculated by “survminer” or the optimal cutoff of gene expression. All patients were divided into Benefit and NonBenefit groups according to the benefit of PD-1/PD-L1 treatment. The statistical difference of lipid score between the two groups was compared by Wilcoxon test.

### Western blot (WB) analysis

Once the cells reached a confluence of approximately 70%-80%, we removed the medium and washed the cells with phosphate-buffered saline (PBS) to eliminate any residual medium. We then lysed the cells with RIPA buffer (radioimmunoassay precipitation solution) containing 1% PMSF (phenylmethylsulfonyl fluoride) (100 mg/ml) on ice, followed by centrifugation of the cell lysate at 12,000 rpm for 10 min to obtain the supernatant. The supernatant was mixed with 5X SDS loading buffer (epizme, China) and heated at 100 °C for 10 min to denature the proteins. Next, we loaded equal amounts of protein samples onto a polyacrylamide gel and performed electrophoresis to separate the proteins. The separated proteins were then transferred onto a PVDF membrane, which was blocked with 5% non-fat milk in TBST (Tris-buffered saline with Tween 20). Subsequently, the membrane was incubated overnight at 4 °C with primary antibodies, namely anti-PTDSS1 (ab237019, Abcam) and anti-vinculin (E1E9V, #13901, CST), followed by washing with TBST. To choose vinculin as the internal reference protein, we considered that the expression of GAPDH might be influenced by metabolism, and tubulin has a molecular weight similar to that of PTDSS1, while vinculin is a cell skeleton-related protein with relatively stable expression. After washing the membrane with TBST, we incubated it with secondary antibodies conjugated with HRP (horseradish peroxidase) (ab6721, Abcam) for 2 h at room temperature. Finally, we visualized the membranes using enhanced chemiluminescence (ECL) plus western blot detection reagents (Thermo Fisher Scientific, USA) and quantified the protein expression levels using densitometry analysis. The relative protein expression was normalized to vinculin.

### EdU assay

EdU assessing kit were bought from Beyotime (Shanghai, China) for the detection of proliferation rate of the mentioned LUAD cells. The experiments were conducted according to the manufacturer’s protocol. In short, a density of 1 × 10^5^ cells/well were determined by cell counting. Then, the cells at such density were seeded in 24-well plates and cultured in the original medium for another day. After incubation with 10 μm EdU reagent for 2 h, the cultured LUAD cells were obtained. 4% paraformaldehyde was utilized for the fixation, which lasted for approximately 15 min. TritonX-100 was used to treat the cells for 10 min, subsequently counterstained with DAPI. Positive cells were identified by fluorescence microscopy (Olympus).

### Colony formation assay

In order to perform the colony formation assay to determine the cell growth status of the LUAD cell line, a density of 500 per well was determined by cell counting. Cells were inoculated in a clean six-well plates and maintained at 37 °C for 10 days. Then, the cells were fixed with 4% paraformaldehyde overnight, dyed with crystal violet. Finally, we counted the cell colonies.

### Flow cytometry

Apoptosis detection using a flow cytometer (BD biosciences, NJ, USA) was conducted in accordance with the specific operation steps of the manufacturer's instructions. The Annexin V-FITC apoptosis detection kit was purchased from Beyotime (shanghai, China). The four quadrants were used to discriminate the early apoptotic cells and late apoptotic cells from the necrotic cells and living cells.

### Statistical and survival analysis

In the TCGA database and GEO validation dataset, we divided the cohort into two groups based on the best cutoff of the score and/or gene expression calculated by R package “survminer”. The Kaplan–Meier curve and log-rank test were constructed using the R package “survival” to evaluate the effect on prognosis. All statistical tests were p < 0.05. SPSS 25.0 software was used for statistical analysis. The quantitative graphing was performed using GraphPad Prism 8.

## Results

### Signature construction of lipid metabolism and survival analysis

**Fig. 1:**
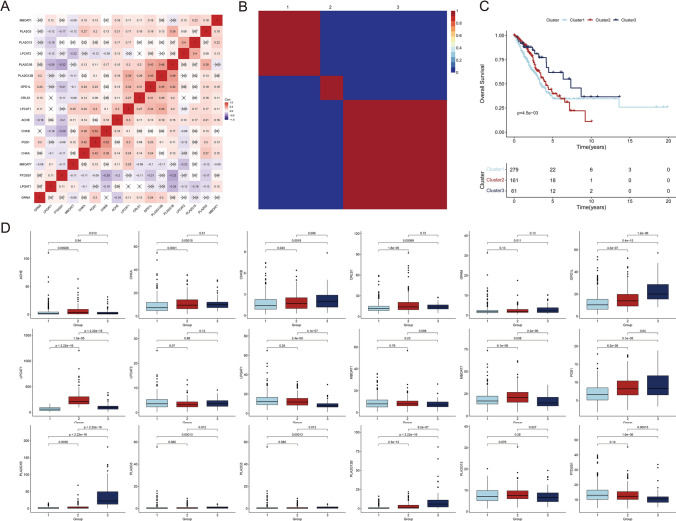
Cluster analysis of lipid metabolism-related genes in LUAD. **A** The co-expression pattern of lipid metabolism-related genes. **B** 510 lung adenocarcinoma samples in TCGA-LUAD were classified into three conserved subtypes according to the lipid metabolism-related genes. **C** Prognostic analysis of three conserved subtypes. **D** The expression of lipid metabolism-related genes in TCGA-LUAD samples

We downloaded 7 dataset of bulk transcriptome data from GSE37745, GSE19188, GSE30219, GSE31547, GSE41271, GSE42127, GSE72094 in GEO database. Single cell dataset of GSE117570 was obtained from TISCH2 database. LogNormalize was used to standardize the data. To construct a lipid metabolism-related gene signature for predicting prognosis in LUAD, we performed a detailed literature review to identify a set of relevant genes (Table S1). The co-expression pattern of these genes was shown in the form of heatmap (Fig. 1A), and the conservative lipid metabolism subtypes were distinguished using non-negative matrix factorization. The best rank was determined to be 3, which divided 510 LUAD samples in TCGA dataset into three conserved subtypes (Fig. 1B). Survival analysis showed that the grouping based on lipid metabolism-related genes had a prognostic effect. Group 3 showed the best prognosis, and groups 1 and 2 showed poor prognosis (Fig. 1C). We further analyzed the molecular characteristics of lipid metabolism in different groups by showing the expression of lipid metabolism genes in LUAD samples using a box plot (Fig. 1D). We compare them in pairs, and the number above the horizontal line represents P value. GPD1L, PLA2G12B, PLA2G3 and PLA2G1B were markedly up-regulated in the group 3, comparing to the group 1 and 2. On the other hand, MBOAT7 and PTDSS1 showed an enhanced expression in group 1 and 2. In addition, LPCAT1 was uniquely elevated in the group 2.

### Prognostic analysis and model construction of lipid-score

**Fig. 2:**
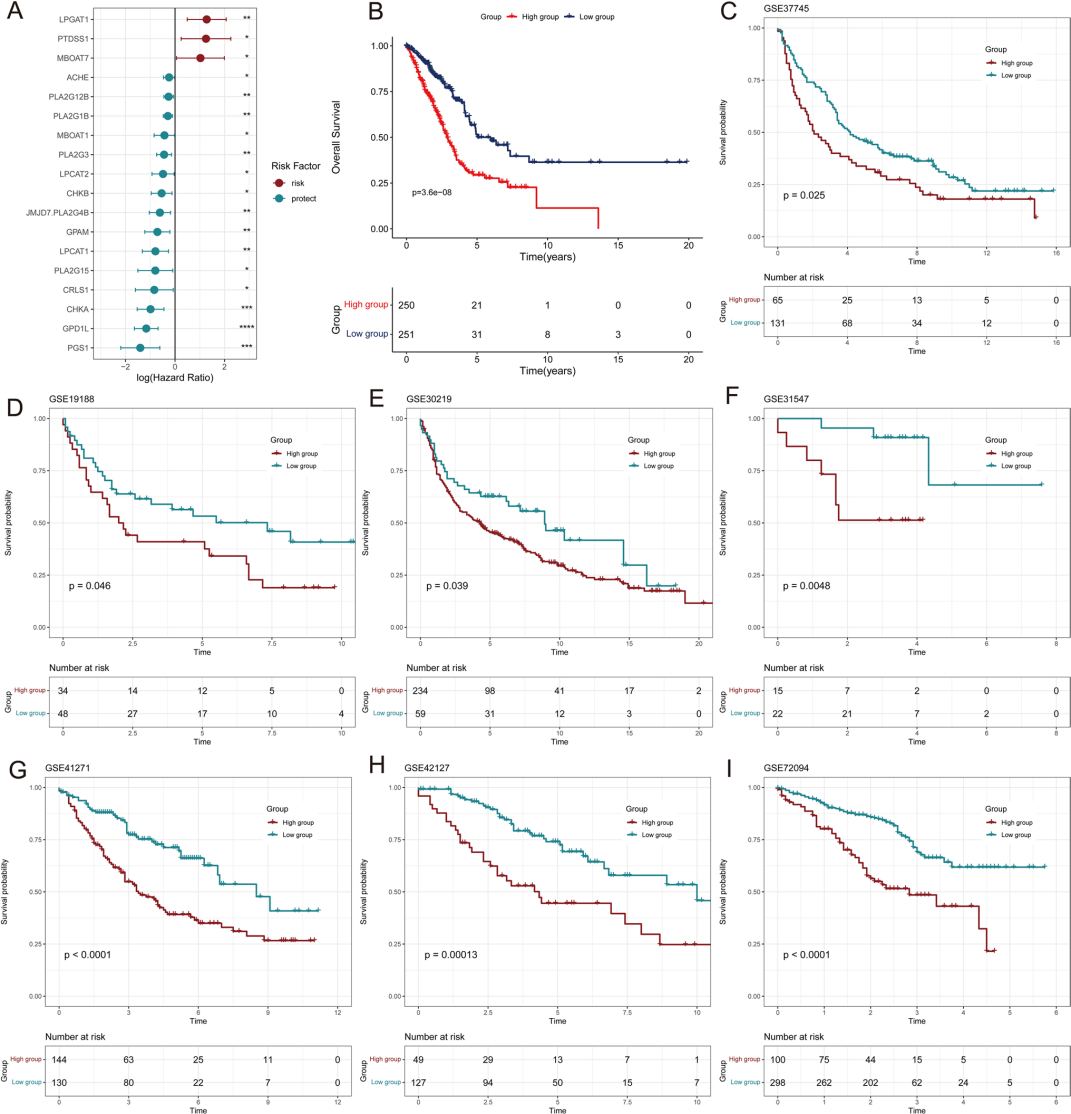
Prognostic analysis and construction of Lipid-score prediction model. **A** Univariate cox regression model. Significant risk factors (red) and protective factors (green) genes were selected to construct the model. The value of Log (Hazard Ratio) was showed in diaphragm. **B** Prognostic analysis of low and high Lipid-score groups. **C**–**I** The verification of Lipid-score in 7 independent validation sets. The p-value were showed in each diaphragm

We performed univariate cox regression on all lipid metabolism-related genes to evaluate their impacts on the prognosis of LUAD patients. Significant risk factors and protective factors were selected for model construction (Fig. 2A). The risk factors included LPGAT1, PTDSS1 and MBOAT7. The protective factors included ACHE, PLA2G12B, PLA2G1B, MBOAT1, PLA2G3, LPCAT2, CHKB, PLA2G4B, GPAM, LPCAT1, PLA2G15, CRLS1, CHKA, GPD1L and PGS1. The GSVA algorithm was used to calculate the score of the samples in TCGA-LUAD based on the above gene expression, named Lipid-score. In TCGA lung adenocarcinoma samples, Lipid-score showed excellent ability to predict prognosis (Fig. 2B). Subsequently, we found that Lipid-score exhibited excellent and consistent prognostic value across seven independent validation sets (GSE37745, GSE19188, GSE30219, GSE31547, GSE41271, GSE42127 and GSE72094) (Fig. 2C–I). These results demonstrate the robust performance of our Lipid-score in predicting the prognosis of LUAD patients.

### Analysis of immune characteristics of lipid-score

**Fig. 3:**
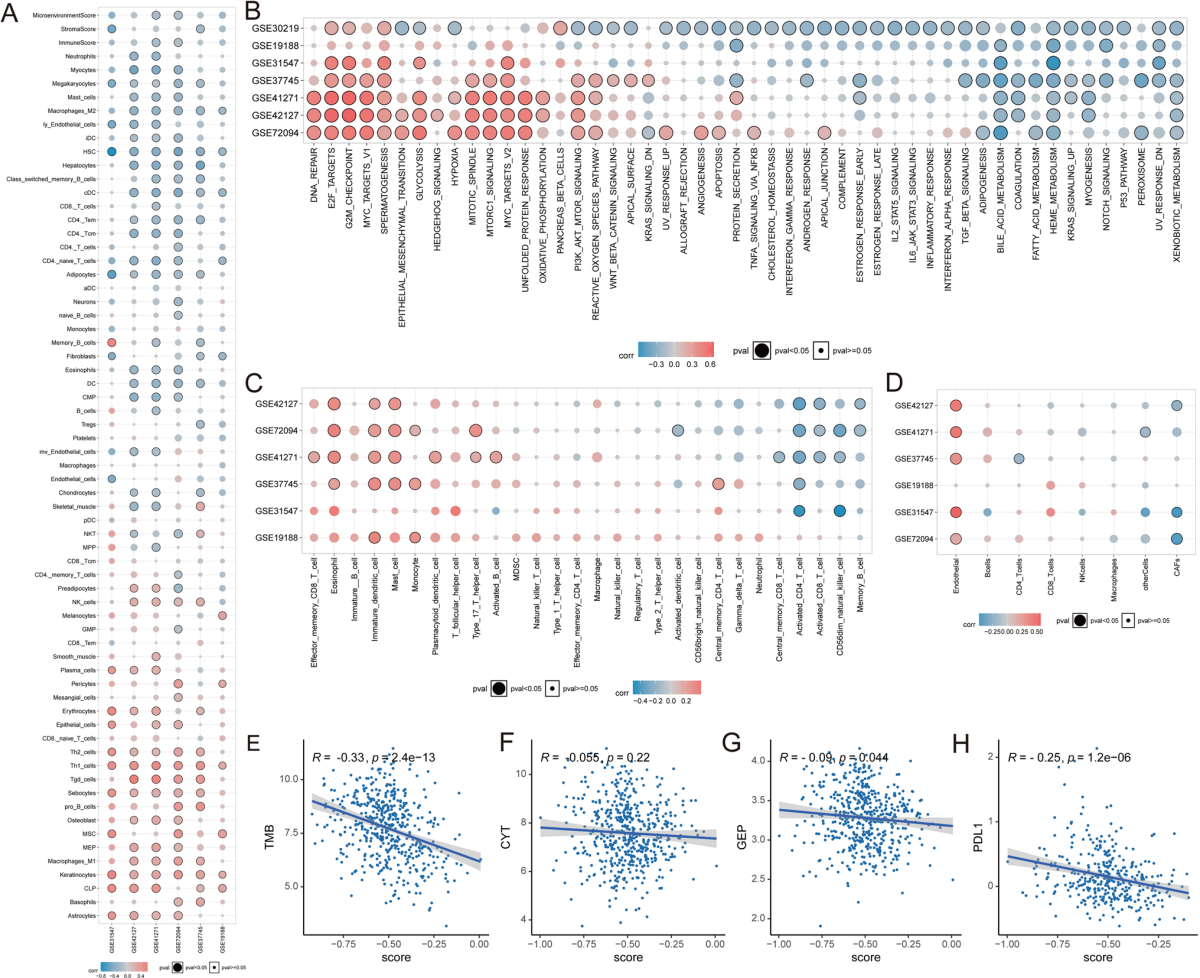
Immune characteristics of Lipid-score. **A**–**D** Enrichment analysis of immune infiltration in validation sets. **E**–**H** Spearman correlation between immunotherapy indicators and Lipid-score

To further elucidate the influences of lipid metabolic signaling on the tumor immune microenvironment, we performed a multidimensional immune cell infiltration analysis. The results showed that M2 macrophages, cDCs (classical DCs), and hematopoietic stem cells (HSCs) were inversely correlated with lipid scores in all groups. We found that Lipid-score exhibited general immunosuppressive efficacy across multiple datasets by using various immune infiltration algorithms (Fig. 3A–D). Specifically, activated CD4 T cells, activated CD8 T cells and CD56 NK cells generally displayed negative correlation with the Lipid-score. Moreover, cancer-associated fibroblasts (CAFs) were also negatively correlated with score and the endothelial cells showed a positive correlation with the score in 5 out of 6 cohorts. The correlation between markers of immunotherapy effect and lipid score showed that high Lipid-scores were mainly negatively related with Tumor Mutation Burden (TMB), gene expression profile (GEP) and PDL1 which implied that the patients with high Lipid-scores may be less likely to benefit from immunotherapy (Fig. 3E–H).

### Drug sensitivity analysis and the predictive effects of lipid-score in immunotherapy

**Fig. 4:**
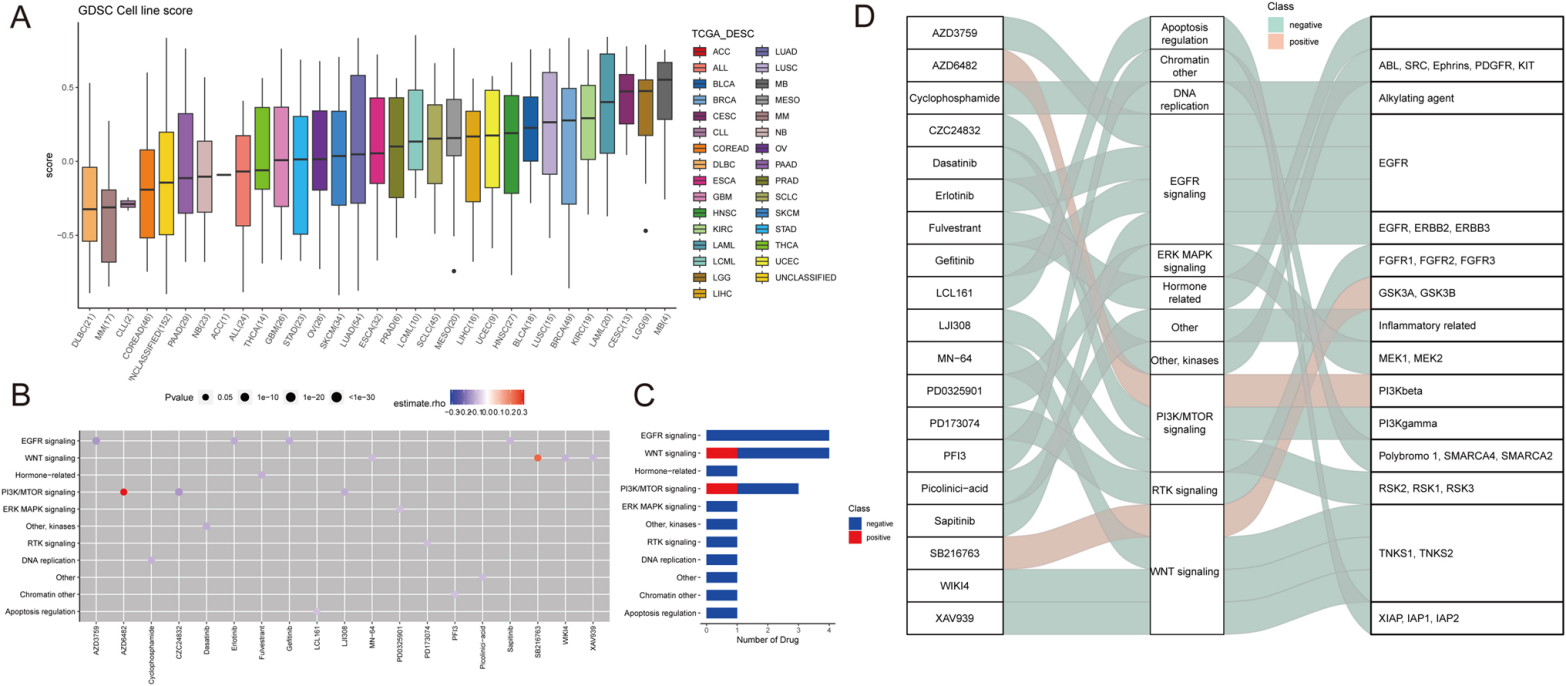
Drug sensitivity analysis based on Lipid-score. **A** The distribution of Lipid-score in different cancer cell lines in the GDSC cancer cell line database. **B**, **C** Drug sensitivity analysis identified a variety of drugs positively correlated with the Lipid-score. **D** Sankey diagram showing drugs and corresponding targets and pathways

**Fig. 5:**
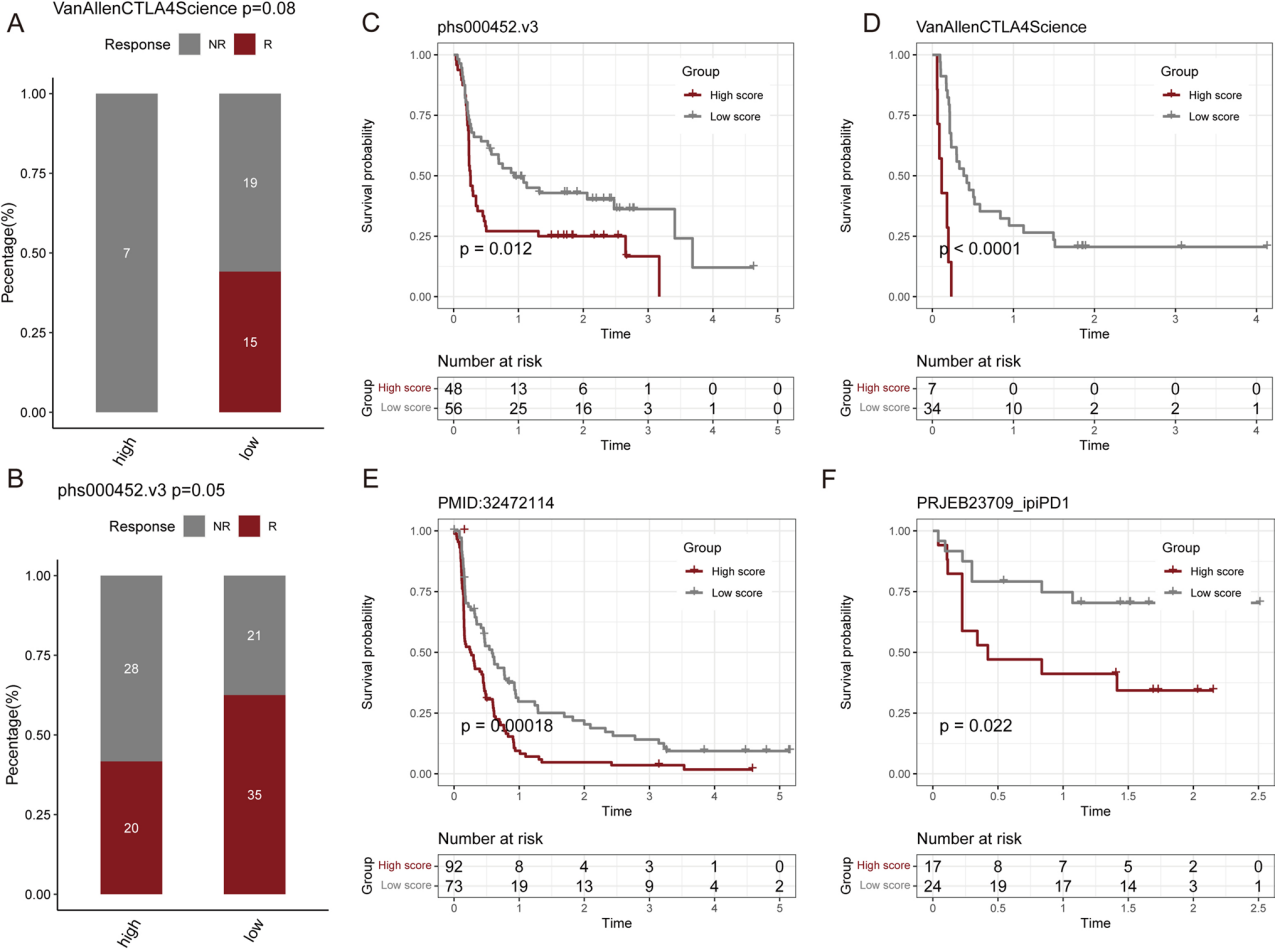
The predictive performances of Lipid-score in immunotherapy. **A**, **B** The distinguishing ability of Lipid-score groups in the response of immunotherapy: Van Allen CTLA4 Science study (**A**) and phs000452 study (**B**). **C**–**F** Prognostic analysis of low and high Lipid-score groups in different immunotherapy cohorts

We identified the distribution of Lipid-score in different cancer cell lines in the GDSC cancer cell line database (Fig. 4A). We performed drug sensitivity analysis based on Lipid-score, and the results revealed a variety of drugs positively correlated with Lipid-score, suggesting the possibility of drug combination (Fig. 4B, C). The drugs and corresponding targets and pathways are shown in Fig. 4D. To validate the predictive performance of Lipid-score on curative effect of immunotherapy, we calculated the Lipid-scores in PMID: 26359337, PMID: 32472114, PRJEB23709, Phs000452 data sets, which all contain data of immunotherapy efficacy. The results demonstrated the ability of Lipid-scores to distinguish the efficacy of immunotherapy (chi-square test, Fig. 5A, B). Based on Lipid-score grouping, the high Lipid-score group showed a lower progression-free survival (Fig. 5C–F).

### Single-cell transcriptome analysis reveals diverse involvement of lipid metabolism pathway signals in LUAD microenvironment

**Fig. 6:**
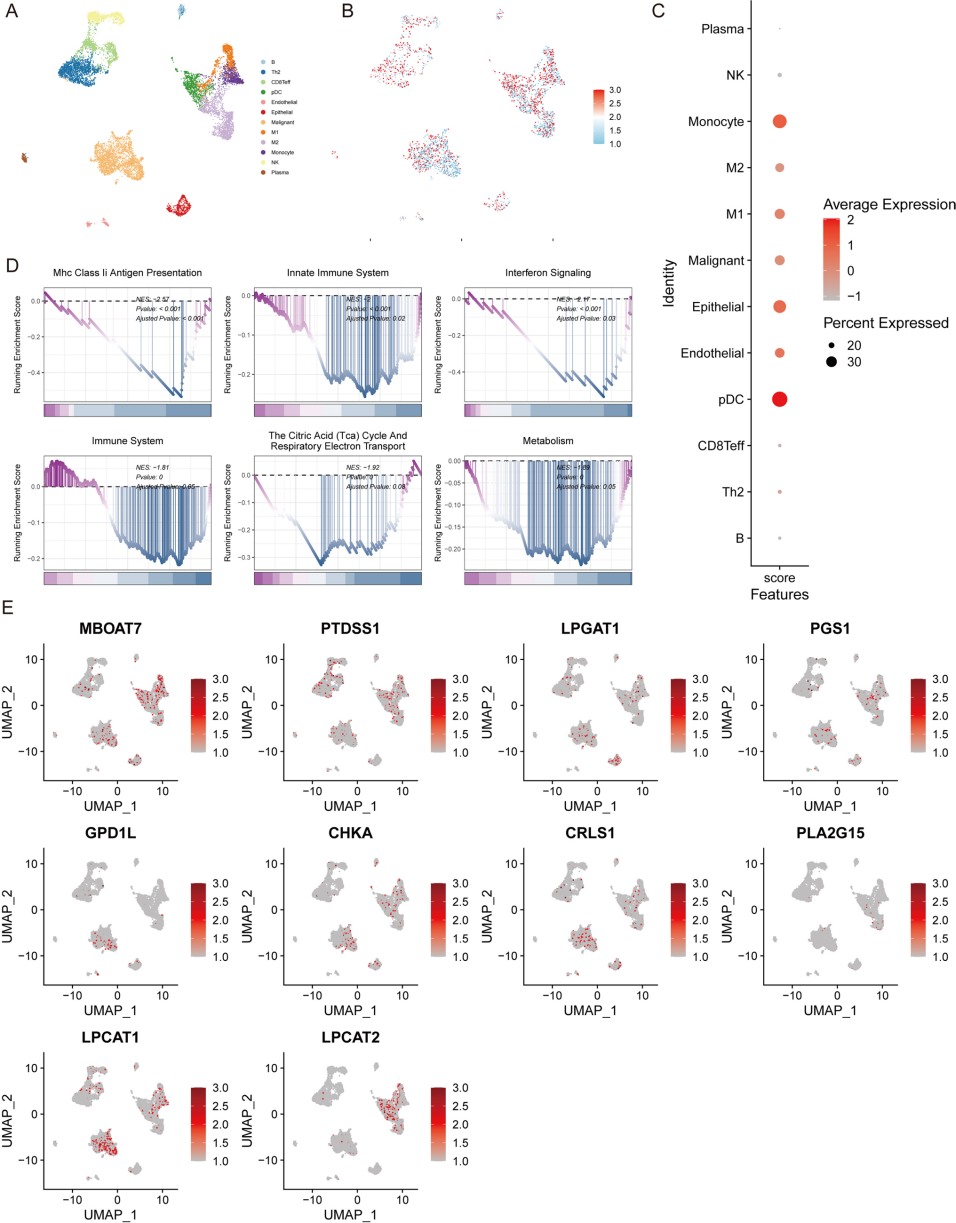
Single-cell transcriptome analysis of Lipid-score in LUAD. **A** The landscape of the cell types in LUAD. **B**, **C** Distribution of Lipid-score in different LUAD cell types. **D** The results of GSEA enrichment analysis. **E** Expressions of marker genes in cell subsets

By utilizing UMAP dimensionality reduction, we annotated malignant cells, stromal cells, and immune cells including B, Th2, CD8 T effector cells, pDCs (plasmacytoid dendritic cells), endothelial cells, epithelial cells, malignant cells, M1 macrophages, M2 macrophages, Monocytes, NK cells, and plasma cells (Fig. 6A). GSVA was used to calculate the distribution of Lipid-score. It was found that the Lipid-score was distributed in various cell types (Fig. 6B), suggesting that lipid metabolism exists in various cells in the tumor microenvironment. The dotplot diagram demonstrated a relatively high expression of Lipid-score in monocytes, epithelial cells and pDCs (Fig. 6C). We further characterized the functions of two groups based on the median of Lipid-score in the single-cell transcriptome dataset. GSEA results revealed the enrichment of Lipid-score in antigen presentation, immune activation, interferon signaling, and other immune pathways (Fig. 6D). The distribution of Lipid-score genes with negative prognostic effects, which is LPGAT1, PTDSS1 and MBOAT7, was particularly evident in malignant cells and macrophages (Fig. 6E).

### Analysis of lipid-score associated intercellular communication processes

**Fig. 7:**
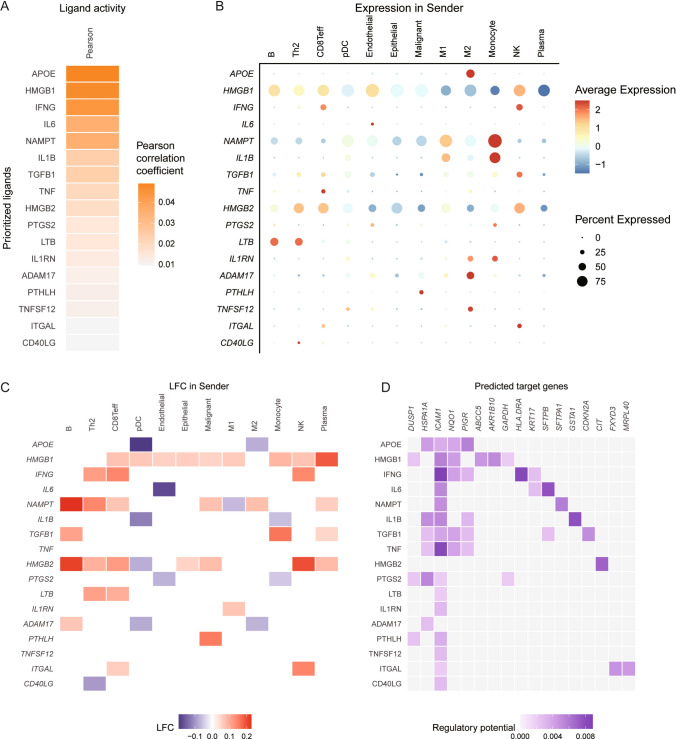
Analysis of intercellular communication processes in different Lipid-score groups. **A** Highly correlated ligand molecules between Lipid-score groups. **B**, **C** Heatmap of the expressions of ligand molecules. **D** The regulatory potential of the downstream predicted genes of ligand molecules

We utilized NicheNet to analyze intercellular communication differences between two groups based on the Lipid-score. We identified ligand molecules with high correlation, among which APOE showed the highest correlation and is closely linked to lipid metabolism (Fig. 7A). These ligands were predominantly expressed in myeloid cells including M2 macrophages and monocytes, suggesting their potential roles as functional carriers of immunosuppression (Fig. 7B, C). Downstream target analysis of these molecules revealed functional information related to Lipid-score mediated immune tolerance and ICAM-related immune cell adhesion (Fig. 7D).

### PTDSS1-knockdown tampered the proliferation and facilitated the apoptosis of LUAD

**Fig. 8:**
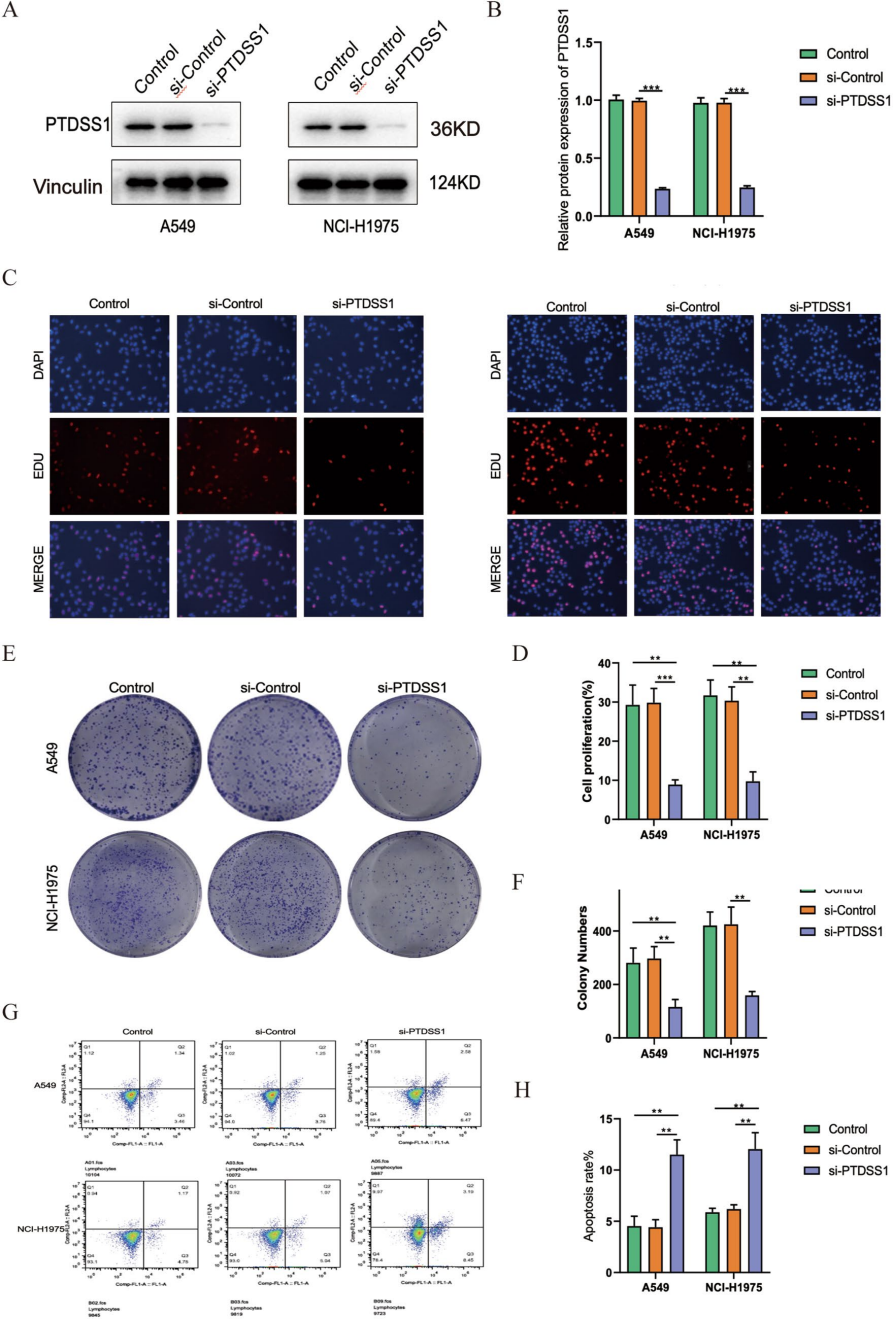
PTDSS1 affected proliferation and apoptosis in LUAD. **A**, **B** Western blot showed that PTDSS1 was successfully knockdown by siRNA. **C**, **D** Edu assays showed that proliferation of A549 and NCI-H1975 cells were inhibited by si-PTDSS1. **E**, **F** Clonal formation assays showed that proliferation of A549 and NCI-H1975 cells were inhibited by si-PTDSS1. **G**, **H** Flow cytometry assessing the LUAD cell apoptosis

The phosphatidylserine synthase 1 (PTDSS1) is a key gene in risk score model. In order to further reveal the potential roles and mechanisms of PTDSS1 in the development of LUAD, we further conducted in vitro experiments. We used A549 and NCI-H1975 cell lines for in vitro experiments. We transfected PTDSS1 siRNA into these cell lines and verified the knockdown effects by applying western blot. We found that PTDSS1 was significantly reduced in siRNA groups (Fig. 8A, B). Edu assays revealed that LUAD cell proliferation was significantly inhibited after reduction of PTDSS1 expression (Fig. 8C, D). Colony formation assays also showed that knockdown of PTDSS1 could inhibit the proliferation of LUAD cells (Fig. 8E, F). Furthermore, we investigated whether PTDSS1 affects apoptosis in LUAD cells. LUAD cells showed a significant increase in apoptotic cells after the knockdown of PTDSS1 (Fig. 8G, H).

## Discussion

LUAD is one of the most lethal cancers that requires a better understanding of its underlying mechanisms to improve patient outcomes [11]. It is well known that metabolic reprogramming is one of the hallmarks of tumorigenesis [12]. Lipid metabolism disorder is one of the important factors affecting metabolic reprogramming and is closely related to the development of cancer [13]. However, robust systematic studies on lipid metabolism in LUAD are still lacking.

The study aimed to construct a lipid metabolism-related risk score model for predicting prognosis and response to immunotherapy in LUAD. We performed a detailed literature review to identify a set of relevant genes and observed the co-expression patterns of these genes. Using NMF, we distinguished the conservative lipid metabolism subtypes, dividing 510 LUAD samples in TCGA datasets into three conserved subtypes. Survival analysis showed that the grouping based on lipid metabolism-related gene signature had a prognostic prediction effects, with group 3 showing the best prognosis, and groups 1 and 2 showing the worst prognosis. The final prognostic score was constructed using Gene Set Variation Analysis (GSVA) and was named Lipid-score, which demonstrated excellent prediction performances of prognosis in TCGA lung adenocarcinoma cohort and seven independent validation sets. Further analysis of immune characteristics of different groups based on Lipid-score revealed high Lipid-score had a general immunosuppressive efficacy and negative correlations with immune cell infiltration. We conducted several analysis targeting pan-immune indicators, which revealed that Lipid-score was negatively correlated with a variety of pan-immune indicators, especially in several immunotherapy efficacy indicators. These results suggested that Lipid-score related genes may play roles in the infiltration of immune cells. We also performed drug sensitivity analysis based on Lipid-score and observed the distribution of Lipid-score in different cancer cell lines in the GDSC cancer cell line database. Drug sensitivity analysis revealed a variety of drugs positively correlated with Lipid-score score, suggesting the possibility of drug combination. In the immunotherapy cohort, Lipid-score demonstrated the ability to distinguish the efficacy of immunotherapy. Based on Lipid-score grouping, the Lipid-score high group showed a poor progression-free survival.

Our study identified three genes, LPGAT1, PTDSS1, and MBOAT7, that are potentially involved in the malignant progression of LUAD. LPGAT1 is a lysophosphatidylglycerol acyltransferase that plays a role in lipid metabolism [14]. In LUAD, LPGAT1 overexpression has been shown to be associated with poor prognosis [15]. This may be due to its involvement in promoting cancer cell proliferation and metastasis, as well as suppressing immune function [16]. Furthermore, LPGAT1 may promote resistance to chemotherapy and targeted therapies, making it a potential target for novel therapeutic approaches [17]. MBOAT7 is as a membrane-bound O-acyltransferase that is involved in lipid metabolism, which overexpression in LUAD could predict poor prognosis [18]. PTDSS1 overexpression is also associated with poor prognosis, and may contribute to the aggressive behaviors of cancer cells by promoting proliferation, migration, and invasion, as well as suppressing immune functions [19]. PTDSS1 expression was reported positively correlated with tumor-associated macrophage (TAM) abundance, while negatively associated with the overall survival of breast carcinoma patients, suggesting PTDSS1 might be developed as a potential therapeutic target to modify tumor-promoting inflammation in breast cancer [20]. Additionally, Wang et al reported that PTDSS1 acts as a oncogene, suggesting that it may be a potential therapeutic target for lung cancer [21]. However, there are still no studies reporting the cellular functions of PTDSS1 in LUAD and its potential as a biomarker, and our study exactly addresses this issue.

This study still has some shortcomings. On the one hand, the Lipid-score was constructed and validated by public datasets, which is still not validated in our own cohort. In the future, we will verify the prognostic value of our constructed lipid score in LUAD in our own cohort. Second, we only explored the effects of PTDSS1 on proliferation and apoptosis. We will explore whether PTDSS1 affects other biological functions of LUAD in further study. Third, we did not conduct cytological studies on MBOAT7 and LPGAT1. We'll explore them in other studies. Finally, this study only explored phenotypes at the bioinformatics level and cellular level. In the next study, we will further investigate the mechanism of lipid metabolism affecting the occurrence and development of LUAD.

In conclusion, the study provides valuable insights into the involvement of lipid-related metabolic genes in predicting prognosis and response to immunotherapy. The results suggested that lipid metabolism plays a role in regulating the immune microenvironment and may be involved in diverse cellular processes in the tumor microenvironment. The findings also suggested the possibility of drug combination and provide potential therapeutic targets for future research. Moreover, we found that knockdown of PTDSS1 significantly tampered LUAD proliferation and facilitated apoptosis.

## Conclusion

Our findings may have important implications for the development of novel strategies to predict response to immunotherapy and prognosis of LUAD. In addition, we investigated the role of PTDSS1 in LUAD carcinogenesis, which showed that PTDSS1 regulated LUAD proliferation and apoptosis.

### Supplementary Information

Below is the link to the electronic supplementary material.

Supplementary file1 (XLSX 17 KB)

## Data Availability

Data can be obtained from the corresponding author when required.

## References

[1] Siegel RL, Miller KD, Wagle NS, Jemal A (2023). Cancer statistics, 2023. CA Cancer J Clin.

[2] Travis WD, Brambilla E, Noguchi M, Nicholson AG, Geisinger K, Yatabe Y, Powell CA, Beer D, Riely G, Garg K, Austin JH, Rusch VW, Hirsch FR, Jett J, Yang PC, Gould M (2011). International association for the study of lung cancer/American thoracic society/European respiratory society: international multidisciplinary classification of lung adenocarcinoma. J Thorac Oncol.

[3] Meza R, Meernik C, Jeon J, Cote ML (2015). Lung cancer incidence trends by gender, race and histology in the United States, 1973-2010. PloS one.

[4] Seguin L, Durandy M, Feral CC (2022). Lung adenocarcinoma tumor origin: a guide for personalized medicine. Cancers.

[5] Cheng C, Geng F, Cheng X, Guo D (2018). Lipid metabolism reprogramming and its potential targets in cancer. Cancer Commun.

[6] Wang G, Qiu M, Xing X, Zhou J, Yao H, Li M, Yin R, Hou Y, Li Y, Pan S, Huang Y, Yang F, Bai F, Nie H, Di S, Guo L, Meng Z, Wang J, Yin Y (2022). Lung cancer scRNA-seq and lipidomics reveal aberrant lipid metabolism for early-stage diagnosis. Sci Transl Med.

[7] Merino Salvador M, Gomez de Cedron M, Moreno Rubio J, Falagan Martinez S, Sanchez Martinez R, Casado E, Ramirez de Molina A, Sereno M (2017). Lipid metabolism and lung cancer. Crit Rev Oncol Hematol.

[8] Zhou H, Chen Y, Xiao Y, Wu Q, Li H, Li Y, Su G, Ke L, Wu J, Li J (2022). Evaluation of the ability of fatty acid metabolism signature to predict response to neoadjuvant chemoradiotherapy and prognosis of patients with locally advanced rectal cancer. Front Immunol.

[9] Wu F, Zhao Z, Chai RC, Liu YQ, Li GZ, Jiang HY, Jiang T (2019). Prognostic power of a lipid metabolism gene panel for diffuse gliomas. J Cell Mol Med.

[10] Zeng D, Ye Z, Shen R, Yu G, Wu J, Xiong Y, Zhou R, Qiu W, Huang N, Sun L, Li X, Bin J, Liao Y, Shi M, Liao W (2021). IOBR: multi-omics immuno-oncology biological research to decode tumor microenvironment and signatures. Front Immunol.

[11] Nasim F, Sabath BF, Eapen GA (2019). Lung cancer. Med Clin North Am.

[12] Hanahan D, Weinberg RA (2011). Hallmarks of cancer: the next generation. Cell.

[13] Bian X, Liu R, Meng Y, Xing D, Xu D, Lu Z (2021). Lipid metabolism and cancer. J Exp Med.

[14] Xu Y, Miller PC, Phoon CKL, Ren M, Nargis T, Rajan S, Hussain MM, Schlame M (2022). LPGAT1 controls the stearate/palmitate ratio of phosphatidylethanolamine and phosphatidylcholine in sn-1 specific remodeling. J Biol Chem.

[15] Gong H, Ma C, Li X, Zhang X, Zhang X, Chen P, Wang W, Hu Y, Huang T, Wu N, Wang X (2023). Upregulation of LPGAT1 enhances lung adenocarcinoma proliferation. Front Biosci (Landmark edition).

[16] Zhang S, Lu Y, Liu Z, Li X, Wang Z, Cai Z (2020). Identification six metabolic genes as potential biomarkers for lung adenocarcinoma. J Comput Biol.

[17] Yu X, Zhang X, Zhang Y (2020). Identification of a 5-gene metabolic signature for predicting prognosis based on an integrated analysis of tumor microenvironment in lung adenocarcinoma. J Oncol.

[18] Saliakoura M, Reynoso-Moreno I, Pozzato C, Rossi Sebastiano M, Galie M, Gertsch J, Konstantinidou G (2020). The ACSL3-LPIAT1 signaling drives prostaglandin synthesis in non-small cell lung cancer. Oncogene.

[19] Yang T, Hui R, Nouws J, Sauler M, Zeng T, Wu Q (2022). Untargeted metabolomics analysis of esophageal squamous cell cancer progression. J Transl Med.

[20] Sekar D, Dillmann C, Sirait-Fischer E, Fink AF, Zivkovic A, Baum N, Strack E, Klatt S, Zukunft S, Wallner S, Descot A, Olesch C, da Silva P, von Knethen A, Schmid T, Grosch S, Savai R, Ferreiros N, Fleming I, Ghosh S, Rothlin CV, Stark H, Medyouf H, Brune B, Weigert A (2022). Phosphatidylserine synthase PTDSS1 shapes the tumor lipidome to maintain tumor-promoting inflammation. Cancer Res.

[21] Wang YT, Lin MR, Chen WC, Wu WH, Wang FS (2021). Optimization of a modeling platform to predict oncogenes from genome-scale metabolic networks of non-small-cell lung cancers. FEBS Open Bio.

